# p130Cas Is Correlated with EREG Expression and a Prognostic Factor Depending on Colorectal Cancer Stage and Localization Reducing FOLFIRI Efficacy

**DOI:** 10.3390/ijms222212364

**Published:** 2021-11-16

**Authors:** Jörg Kumbrink, Pan Li, Agnes Pók-Udvari, Frederick Klauschen, Thomas Kirchner, Andreas Jung

**Affiliations:** 1 Faculty of Medicine, Institute of Pathology, Ludwig-Maximilians-University of Munich, 80337 Munich, Germany; s-pali@helios.med.uni-muenchen.de (P.L.); udvari.agnes@gmail.com (A.P.-U.); Frederick.Klauschen@med.uni-muenchen.de (F.K.); Thomas.Kirchner@med.uni-muenchen.de (T.K.); Andreas.Jung@med.uni-muenchen.de (A.J.); 2 German Cancer Consortium (DKTK), Partner Site Munich, 80336 Munich, Germany

**Keywords:** p130Cas, BCAR1, EREG, colorectal cancer, therapy resistance, FOLFIRI, metastasis

## Abstract

p130 Crk-associated substrate (p130Cas) is associated with poor prognosis and treatment resistance in breast and lung cancers. To elucidate p130Cas functional and clinical role in colorectal cancer (CRC) progression/therapy resistance, we performed cell culture experiments and bioinformatic/statistical analyses of clinical data sets. p130Cas expression was associated with poor survival in the cancer genome atlas (TCGA) data set. Knockdown/reconstitution experiments showed that p130Cas drives migration but, unexpectedly, inhibits proliferation in CRC cells. TCGA data analyses identified the growth factor epiregulin (EREG) as inversely correlated with p130Cas. p130Cas knockdown and simultaneous EREG treatment further enhanced proliferation. RNA interference and EREG treatment experiments suggested that p130Cas/EREG limit each other’s expression/activity. Inverse p130Cas/EREG Spearman correlations were prominent in right-sided and earlier stage CRC. p130Cas was inducible by 5-fluorouracil (5-FU) and FOLFIRI (folinic acid, 5-FU, irinotecan), and p130Cas and EREG were upregulated in distant metastases (GSE121418). Positive p130Cas/EREG correlations were observed in metastases, preferentially in post-treatment samples (especially pulmonary metastases). p130Cas knockdown sensitized CRC cells to FOLFIRI independent of EREG treatment. RNA sequencing and gene ontology analyses revealed that p130Cas is involved in cytochrome P450 drug metabolism and epithelial-mesenchymal transition. p130Cas expression was associated with poor survival in right-sided, stage I/II, MSS (microsatellite stable), or BRAF-mutated CRC. In summary, p130Cas represents a prognostic factor and potential therapeutic target in CRC.

## 1. Introduction

Colorectal cancer (CRC) is the third most common cancer in men and second most common in women, with about 1.85 million people affected globally, accounting for 9.2% of all cancer deaths [1]. The high mortality is explained in part because nearly 20% of patients present with metastatic disease and 25–30% of patients with stage II/III disease experience recurrence within five years of a curative intent surgery [2]. The development of drug resistance is another major obstacle in the treatment of metastatic CRC (mCRC) [3]. Moreover, the high mortality rate of mCRC patients (5-year survival of only 12.5%) illustrates the need for further elucidating the mechanisms of CRC progression and therapy resistance and to identify novel prognostic markers and potential therapeutic targets for CRC [4].

CRC development and progression are characterized by the sequential accumulation of epigenetic and genetic changes described as the adenoma-carcinoma sequence [5]. The initiation event is commonly a mutation in the adenomatous polyposis coli (*APC*) tumor suppressor gene leading to increased Wnt/β-catenin signaling, followed by subsequent mutations in the Kirsten rat sarcoma viral proto-oncogene (*KRAS*), tumor protein p53 (*TP53*) genes, and a frequent inactivation of the TGF-β signaling pathway often by mutations in the mothers against decapentaplegic (*SMAD4*) gene. More recently, the consensus molecular subtype (CMS) classification was established, which is majorly based on gene expression signatures but also includes genetic, epigenetic, proteomic, and biological/metabolic features of primary tumors [6]. Four CMS groups and their prognostic relevance were defined: CMS1 (microsatellite instability (MSI) immune), CMS2 (canonical), CMS3 (metabolic) and CMS4 (mesenchymal). In the following years, the CMS paradigm was also translated into the context of mCRC [7,8,9]. These and other studies showed that Wnt/β-catenin, KRAS/mitogen-activated protein kinase (MAPK), and phosphatidylinositol 3-kinase/AKT serine-threonine protein kinase (PI3K/AKT) signaling pathways are commonly deregulated in CRC [10,11].

One signaling molecule that integrates and thus controls the aforementioned signaling pathways is p130 Crk-associated substrate (p130Cas) [12,13,14]. p130Cas, encoded by the breast cancer anti-estrogen resistance 1 (*BCAR1*) gene [15], is a scaffold protein that integrates large multi-protein complexes in response to stimuli such as hormones, growth factors, and integrin engagement. Upon environmental signals, Src family members and focal adhesion kinase (FAK) phosphorylate the p130Cas substrate domain (SD). This active p130Cas can recruit adaptor proteins such as Crk and Nck to its phosphorylated SD [16]. These complexes are involved in the regulation of KRAS/MAPK, PI3K/AKT, Wnt/β-catenin, and c-Jun N-terminal kinase (JNK) signaling pathways controlling cell survival, cell cycle regulation, proliferation, adhesion, migration, and invasion [13,14,17]. These cellular programs are commonly altered in various malignancies [18], indicating the importance of a tightly controlled p130Cas signaling.

Knowledge on *BCAR1*/p130Cas function and clinical association is mainly based on studies in mammary carcinomas [13,14]. In the past decade, researchers expanded their studies and could show the importance of p130Cas in tumor progression and metastasis in other cancer entities, such as prostate carcinoma [19], lung adenocarcinoma [20], pancreatic cancer [21], and oral squamous cell carcinoma [22], too. Only a few studies addressed its role in CRC. We previously identified a potential association of *BCAR1* expression with CRC [23]. Other studies showed the involvement of p130Cas in α1-integrin/c-Src-mediated invasion of CRC cells [24], in c-Src regulated PI3K/AKT-driven colon tumorigenesis [25,26], and cysteine-rich protein 2 (CSRP2) controlled suppression of CRC progression [27].

In this study, we show that *BCAR1*/p130Cas drives migration and inhibits proliferation in CRC cells by limiting the expression of the epidermal growth factor receptor (EGFR) ligand epiregulin (EREG). p130Cas and EREG control each other’s expression/activity, and their expression is correlated inversely in early-stage and right-sided tumors and positively in late-stage cancer and metastasis. p130Cas but not EREG mediates a reduced response to FOLFIRI and is associated with poor outcomes in distinct CRC subgroups. Thus, *BCAR1*/p130Cas represents a prognostic marker and potential therapeutic target in CRC.

## 2. Results

### 2.1. BCAR1 Is Strongly Expressed and Associated with Poor Survival in Colorectal Cancer

**Figure 1:**
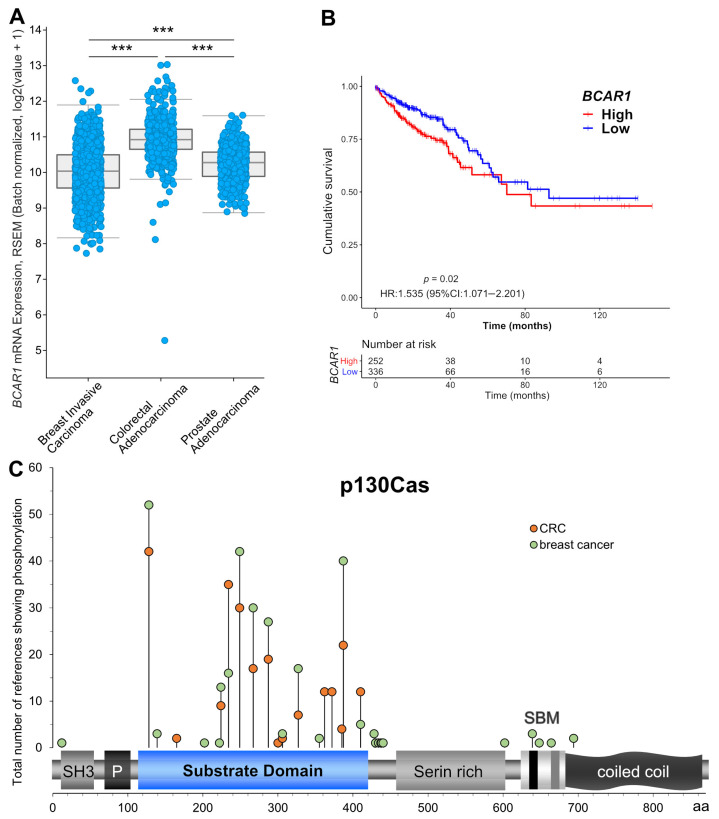
*BCAR1*/p130Cas is strongly expressed in and associated with reduced survival of colorectal cancer patients (TCGA data set). (**A**) *BCAR1* expression was compared in the indicated TCGA PanCancer Atlas data sets by Kruskal–Wallis test (*** *p* < 0.0001). (**B**) Overall survival (OS) of patients of the TCGA CoAd cohort expressing high (red curve) or low (blue curve) *BCAR1* levels. HR, hazard ratio. CI, confidence interval. (**C**) Number of studies showing phosphorylation of certain motifs in the different p130Cas domains in CRC and breast cancer as curated at PhosphositePlus. P, proline-rich domain. SBM, bipartite Src binding motif.

In a first step, we aimed to elucidate whether *BCAR1*/p130Cas is associated with progression and therapy resistance of CRC. Therefore, we investigated available TCGA (the cancer genome atlas) data sets retrieved from cBioportal [28]. *BCAR1* expression was significantly higher (*p* < 0.001) in colon adenocarcinoma (CoAd) than in prostate adenocarcinoma and breast invasive carcinoma (Figure 1A). These cancer entities were chosen as expression reference as p130Cas was previously identified as overexpressed and as an unfavorable clinical marker in breast and prostate cancer [13,19]. This even higher expression in CoAd suggested a clinical role in CRC as well. Indeed, higher *BCAR1* levels were significantly associated with reduced survival in the CoAd cohort (*BCAR1* high: *n* = 252; *BCAR1* low: *n* = 336; *p* = 0.02, HR = 1.535 (95%CI 1.071–2.201)) (Figure 1B). Genetic alterations are commonly related to cancer development and aggressiveness [29]. However, *BCAR1* alteration frequencies were low in TCGA data sets of 13 different tumor entities (average: 0.9%; CoAd: 1.5%), and no hotspot mutation region was found (Figure S1A–C).

A PhosphoSitePlus [30] analysis revealed that the same hotspot motifs (Y128, Y234, Y249, Y387) as in breast cancer were also phosphorylated in CRC cell lines and tissues (Figure 1C). Moreover, certain motifs were reported as phosphorylated more often (Y234: 2.2-fold; Y410: 2.4-fold) in CRC than in breast cancer or phosphorylated in CRC (Y362 and Y372 each 12 times) but not in breast cancer. These results suggest that enhanced p130Cas expression and phosphorylation but not altered function/activity due to gene mutations may be involved in CRC progression and aggressiveness.

### 2.2. p130Cas Is Strongly Expressed and Phosphorylated and Drives Migration but Inhibits Proliferation in CRC Cell Lines

**Figure 2:**
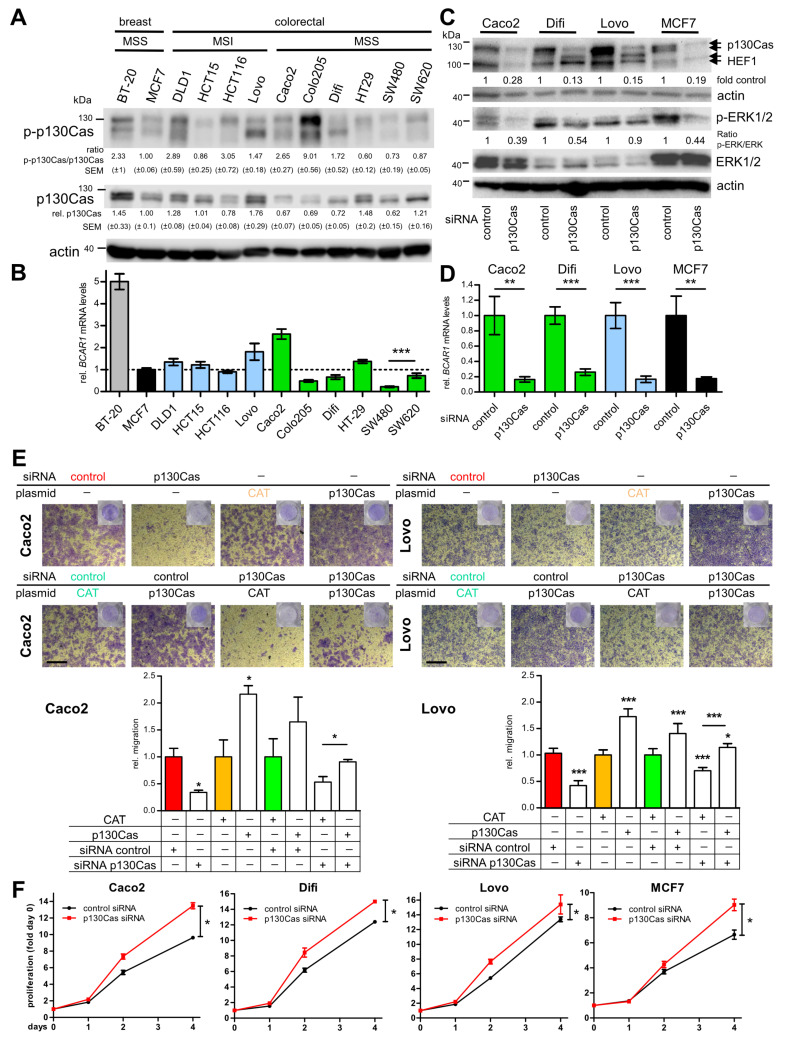
p130Cas is active, strongly expressed, and drives migration but inhibits proliferation in CRC cell lines. (**A**,**B**) Active p130Cas (phosphorylated, p-p130Cas) and p130Cas protein (**A**) and mRNA (**B**) levels in CRC and breast cancer cell lines. Cells were seeded at 1 × 10^5^ cells/well in 6-well plates and cultured in regular medium for 48 h. (**A**) Expression and phosphorylation (p-p130Cas, pooled phospho-antibodies) in whole-cell extracts (WCE, 20 µg) were investigated by immunoblot (IB). Band intensities were quantified by densitometric analysis. The ratio of p-p130Cas and p130Cas was calculated and related to MCF7 levels (set to 1). Average values from three independent experiments are shown. (**B**) mRNA expression in indicated cell lines was normalized to MCF7 levels (dashed line). (**C**,**D**) Cells were transfected with siRNA targeting p130Cas or control siRNA and harvested at 48 h. Knockdown of p130Cas, as well as reduced ERK1/2 phosphorylation, were confirmed by IB (**C**) and qPCR (**D**, *BCAR1* only). (**E**) Cells transiently transfected with p130Cas or CAT-control plasmids and indicated siRNAs were subjected to serum-stimulated migration assay. Upper panel, shown are representative images from at least two independent experiments. Scale bars, 500 µm. Lower panel, shown are fold migration (controls (CAT, siRNA control or CAT+control siRNA) were set to 1) and SEM of triplicates. (**F**) Proliferation assay after p130Cas knockdown was performed as described in Materials and Methods. Statistical differences between growth curves were calculated with the CGGC permutation test [31]. Ns, not significant. * *p* < 0.05. ** *p* < 0.01. *** *p* < 0.001.

In the next step, we investigated possible underlying molecular mechanisms. Active (phosphorylated) p130Cas is a key driver of migration and control of proliferation in breast cancer [14]. Therefore, we initially investigated p130Cas expression and phosphorylation levels in a panel of CRC cell lines (microsatellite instable (MSI) *n* = 4; microsatellite stable (MSS *n* = 6)) with different mutational statuses (Table S2). Additionally, two breast cancer cell lines with known intermediate (MCF7) and high (BT-20) p130Cas expression and phosphorylation were examined to classify the levels in CRC cell lines (Figure 2A and Figure S2A,B). p130Cas protein expression ranged from 0.62 to 1.76-fold compared with MCF7 (BT-20 1.45-fold). Three CRC cell lines showed similar levels (0.75–1.25-fold), four displayed slightly lower (0.62–0.72-fold), and three had higher levels than MCF7. The phosphorylation ratio was similar in two, somewhat lower in two (0.60–0.73-fold) and higher in six CRC cell lines. Four cell lines (2.65–9.01-fold) even exceeded BT-20 phosphorylation levels (2.33-fold). However, no correlation of expression or phosphorylation strength with MSI or mutational status was found. Similar results, although not always correlating with protein levels, were observed on mRNA expression (Figure 2B). Interestingly, protein and mRNA levels were significantly increased in cells isolated from a metastasis (SW620) compared with primary tumor cells (SW480) from the same donor.

To investigate whether active p130Cas also has a functional role in CRC cells, *BCAR1*/p130Cas expression was knocked down by RNA interference (RNAi), and subsequently, transfectants were analyzed by transwell migration assays. Transient knockdown by pooled short-infering RNA (siRNA) was performed in three CRC (Caco2, Difi, Lovo) and MCF7 breast cancer (as control) cell lines. Downregulation was confirmed by Western blot (Figure 2C), immunofluorescence (Figure S2C), and RT qPCR (Figure 2D). To initially test for functional effects of p130Cas knockdown, the phosphorylation levels of the downstream targets ERK1/2 were analyzed (Figure 2C). Expectedly, p130Cas siRNA treatment resulted in a strong reduction in ERK1/2 phosphorylation in Caco2 (50–70%), Difi (40–60%), and MCF7 (50–70%) and a moderate influence in Lovo (10–20%) cells (Figure 2C). Next, migration assays showed that p130Cas knockdown significantly reduced the migratory potential in Caco2 (0.62-fold control transfected), Difi (0.54-fold), Lovo (0.71-fold), and MCF7 (0.8-fold) cells (Figure S3A). Conversely, p130Cas overexpression significantly enhanced migration in Caco2 (2.2-fold) and Lovo (1.72-fold) cells (Figure 2E). Finally, p130Cas reconstitution experiments in siRNA-treated cells were conducted (Figure 2E and Figure S3B). Migration was restored to normal levels in Caco2 (from 0.53 to 0.92) and in Lovo (from 0.7 to 1.14) cells, further indicating a direct regulation of migration by p130Cas in CRC cell lines.

Since p130Cas is also involved in the control of proliferation [12], proliferation assays were conducted with p130Cas or control siRNA transfected Caco2, Difi, Lovo, and MCF7 cells (Figure 2F). In our approach, a significant increase in proliferation was observed in all four cell lines after knockdown of p130Cas, which occurred slightly earlier in CRC than in MCF7 breast cancer cells. Taken together, our results indicate that active p130Cas is strongly expressed in CRC cells and drives migration but inhibits proliferation in these cells.

### 2.3. p130Cas/BCAR1 and EREG Expression Is Negatively Correlated in Colorectal Adenocarcinomas and CRC Cell Lines

**Figure 3:**
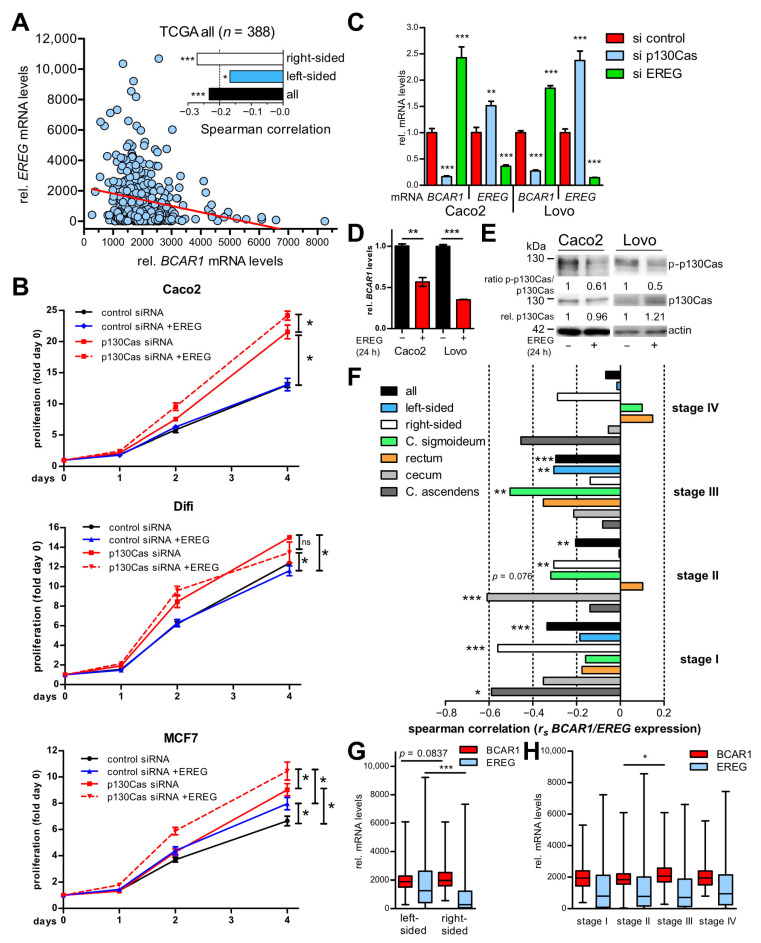
p130Cas/*BCAR1* and *EREG* expression are negatively correlated in CRC cell lines and in right-sided and earlier stages of colorectal adenocarcinomas. (**A**,**F**–**H**) *BCAR1* and *EREG* mRNA levels in patient samples of the TCGA CoAd data set were analyzed. Patient numbers for each investigated subgroup are presented in Table S3A. (**A**,**F**) *BCAR1*/*EREG* expression correlation calculated with Spearman correlation in the indicated subgroups. Spearman coefficients (*r_s_*) are shown. (**B**) Cells were transfected with control or p130Cas siRNA and subsequently incubated with or without recombinant EREG (12.5 ng/mL) or vehicle control. Proliferation assays were performed as described in Materials and Methods. Statistical differences between growth curves were calculated with the CGGC permutation test. (**C**) Cells were transfected with control, p130Cas, or EREG siRNA. *BCAR1* and EREG mRNA expression was quantified with RT qPCR. Shown are expression levels in relation to control siRNA (set to 1) from one representative out of three performed experiments. (**D**,**E**) Cells were treated with recombinant EREG (12.5 ng/mL) or vehicle control for 24 h. (**D**) *BCAR1* mRNA expression was quantified with RT qPCR. (**E**) Expression and phosphorylation (p-p130Cas, pooled phospho-antibodies) in WCE (20 µg) was investigated by IB. Band intensities were quantified by densitometric analysis. The ratio of p-p130Cas and p130Cas was calculated and related to vehicle control (set to 1). (**A**–**H**) ns, not significant. * *p* < 0.05. ** *p* < 0.01. *** *p* < 0.001. si, short-interfering RNA.

As p130Cas is an integral part of a regulatory network, we wanted to identify factors involved in proliferation that might be regulated by p130Cas in CRC cells. Thus, we analyzed the TCGA CoAd data set for gene expressions positively or negatively correlated with *BCAR1* levels. We focused on genes that are related to epidermal growth factor receptor (EGFR) signaling because EGFR is a well-known driver of proliferation and is, therefore, a target for EGFR-directed antibody treatment in mCRC [32]. Our analysis revealed a moderate but significant negative correlation (Spearman’s correlation coefficient (*r_s_* = −0.233, *p* < 0.0001)) of *BCAR1* and *Epiregulin* (*EREG*), an EGFR ligand, in the whole TCGA patient collective (*n* = 388) spanning American Joint Committee on Cancer (AJCC) stages I to IV and left- and right-sided tumors (Figure 3A).

To investigate the influence of EREG on p130Cas-mediated growth inhibition, proliferation assays were performed with cells transiently transfected with p130Cas or control siRNA that were subsequently treated with recombinant EREG (Figure 3B). As observed before, p130Cas knockdown increased proliferation in all cell lines. The addition of EREG further significantly stimulated proliferation in Caco2 and MCF7 cells but not in Difi cells. Interestingly, EREG application did not influence proliferation in control siRNA transfected CRC cells but increased proliferation in MCF7 cells also in the control transfected setting. These results suggested that p130Cas may not only reduce proliferation itself but also impede EREG-mediated cell growth in CRC cells.

To understand the interrelationship between p130Cas and EREG RNAi, experiments were conducted. CRC cell lines and MCF7 cells were transfected with p130Cas siRNA. Knockdown of p130Cas resulted in significantly increased *EREG* expression (Figure 3C and Figure S4A). Conversely, reduction of *EREG* by RNAi led to significantly enhanced *BCAR1* levels (Figure 3C). Treatment with recombinant EREG reduced *BCAR1* mRNA levels but did not majorly influence p130Cas protein expression (Figure 3D,E). However, p130Cas phosphorylation was greatly decreased at 24 h of EREG treatment (Figure 3E). These results suggest that p130Cas and EREG limit each other’s expression/activity.

### 2.4. The Negative Correlation of BCAR1 and EREG Expression Is More Pronounced in Right-Sided and Earlier Stages of Colorectal Adenocarcinomas

EREG expression is associated with suitable outcome especially in left-sided mCRC [33,34]. Therefore, a comprehensive *BCAR1/EREG* expression correlation analysis of the TCGA CoAd data set distinguishing tumor localization and stage was conducted (Figure 3A,F–H, Table S3A). The *r_s_* in left-sided (*n* = 197) and right-sided (*n* = 191) tumors was −0.1674 (*p* = 0.0187) and −0.2710 (*p* = 0.0001), respectively (Figure 3A,F). This stronger negative correlation in right-sided tumors was supported by significantly lower *EREG* levels in right-sided tumors (52% lower, *p* < 0.0001) accompanied by slightly enhanced *BCAR1* levels (7% higher, *p* = 0.0837) (Figure 3G). Further tumor sub-classification showed significant negative *BCAR1*/*EREG* correlations in AJCC stages I (*r_s_* = −0.3355; *p* = 0.0005), II (*r_s_* = −0.2045; *p* = 0.0024) and III (*r_s_* = −0.2951; *p* = 0.0001) whereas no correlation was observed in the advanced stage IV tumors (*r_s_* = −0.0672; *p* = 0.5411) (Figure 3E, Table S3A). Nevertheless, total *EREG* levels were similar in all stages (*p* = 0.5711) and only a slight but significant increase (10%, *p* < 0.05) in *BCAR1* levels was observed in stage III tumors compared with stage II (Figure 3H). Additional partitioning according to localization revealed the strongest negative correlation in right-sided stage I tumors (*r_s_* = −0.5603; *p* = 0.0006), which was majorly accredited to C. ascendens originated cancers (*r_s_* = −0.5893; *p* = 0.0208). Right-sided tumors had stronger inverse correlations than left-sided tumors in all stages except stage III (left-sided: *r_s_* = −0.3041; *p* = 0.0034; right-sided: *r_s_* = −0.1383; *p* = 0.3434). In stage III the strongest *r_s_* values were found in C. sigmoideum samples (*r_s_* = −0.5042; *p* = 0.0017). In contrast, in stage IV no significant correlation and rather a trend to a positive *BCAR1*/*EREG* correlation was observed in left-sided tumors. However, right-sided tumors still displayed inverse but not significant *r_s_* values (C. ascendens: *r_s_* = −0.4545; *p* = 0.1912) at stage IV.

These findings show a more pronounced negative correlation of *BCAR1* and *EREG* expression in right-sided and earlier CRC stages. Nevertheless, a disconnection of *BCAR1* and *EREG* in late-stage left-sided tumors point to the involvement of other factors/mechanisms influencing their expression.

### 2.5. BCAR1 and EREG Expression Is Induced by Standard Chemotherapy and Upregulated in Distant Metastases

**Figure 4:**
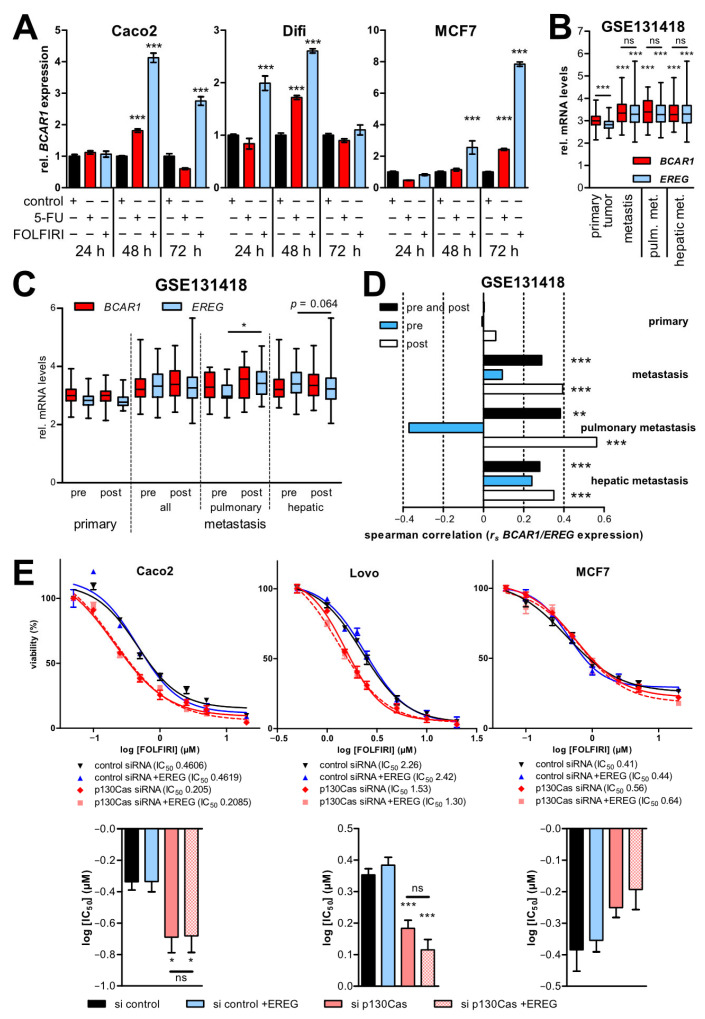
p130Cas/*BCAR1* and *EREG* expression are upregulated in distant metastases, and *BCAR1* downregulation sensitizes CRC cells to FOLFIRI. (**A**) Cells were seeded in 6-well plates and treated after 1 d with vehicle control, 5-FU (20 µM) or FOLFIRI (20 µM 5-FU) for indicated times. *BCAR1* mRNA expression was quantified with RT qPCR. Shown are expression levels in relation to vehicle control (set to 1) from one representative out of three performed experiments. (**B**–**D**) *BCAR1* and *EREG* levels in patient samples of the GSE131418 data set were analyzed. Patient numbers for each investigated subgroup are presented in Table S3B. (**C**) *BCAR1* and *EREG* expression in pre- and post-treatment patient samples. (**D**) *BCAR1*/*EREG* expression correlation calculated with Spearman correlation. Spearman coefficients (*r_s_*) are presented. (**E**) Cells were transfected with control or p130Cas siRNA and subsequently incubated with or without recombinant EREG (12.5 ng/mL) and increasing concentrations of FOLFIRI. Cell viability was measured with AlamarBlue assays. Shown are results from one representative out of three performed experiments. IC_50_ values are indicated. Lower panels, significant differences between response curves were estimated by one-way ANOVA and Bonferroni’s multiple comparison post-test approach. (**A**–**E**) ns, not significant. * *p* < 0.05. ** *p* < 0.01. *** *p* < 0.001. IC50, half-maximal inhibitory concentration.

Since p130Cas is induced by anti-estrogen therapy [35] and doxorubicin [36] in breast cancer cells, the effects of standard CRC chemotherapy on p130Cas/*BCAR1* and *EREG* expression were investigated in CRC cell lines and metastasis tissues samples. First, Caco2, Difi, and MCF7 cells were treated with 5-fluorouracil (5-FU), FOLFIRI (folinic acid, 5-FU, irinotecan) or vehicle control for up to 72 h, and *BCAR1* levels were analyzed by qPCR (Figure 4A). *BCAR1* expression was significantly upregulated by both treatments in all tested cell lines. However, the influence of FOLFIRI was 1.5–3.1-fold stronger than that of 5-FU. The highest increase in Caco2 (4.12-fold) and Difi (2.6-fold) cells was observed at 48 h by FOLFIRI treatment followed by a reduction to 2.75-fold in Caco2 and to control levels (1.1-fold) in Difi cells at 72 h. Notable, a stronger and sustained induction by FOLFIRI was found in MCF7 cells, reaching its maximum of 7.85-fold at 72 h.

Second, *BCAR1* and *EREG* expression correlations in primary tumors and metastases pre and post standard treatment were analyzed employing expression data from the GSE121418 data set. Similar to TCGA results, in primary tumors, *BCAR1* expression was significantly higher than *EREG* levels (Figure 4B). However, no significant correlation of *BCAR1*/*EREG* expression was found (Figure 4C). Further distinction according to CRC stages was not possible due to missing stage information. Compared with primary tissues, in metastases samples, a significant increase in both *BCAR1* and *EREG* levels was observed (Figure 4B). Moreover, similar levels of both genes were found in metastases, which were associated with a moderate positive correlation of *BCAR1*/*EREG* expression (*r_s_* = 0.29; *p* < 0.0001) (Figure 4D, Table S3B). Interestingly, the positive correlation was more pronounced in pulmonary (*r_s_* = 0.38; *p* = 0.011) vs. hepatic (*r_s_* = 0.28; *p* = 0.0008) metastases. No correlation was observed in pre-treated tissues (*r_s_* = 0.094; *p* = 0.49) whereas a positive correlation (*r_s_* = 0.394; *p* < 0.0001) was found in post-treatment tissues. The strongest therapy-induced co-expression of *BCAR1*/*EREG* was determined in pulmonary metastasis (pre: *r_s_* = −0.37; *p* = 0.24; post: *r_s_* = 0.563; *p* = 0.0001), which was accompanied by a numerical upregulation of *BCAR1* and a significant increase in *EREG* levels (Figure 4C). No therapeutic influence on either *BCAR1*/*EREG* expression or correlations was found in primary CRC tissues. These results show that *BCAR1* and *EREG* expression is induced by standard chemotherapy and upregulated in distant metastases, especially in pulmonary metastases.

### 2.6. p130Cas Knockdown Sensitizes CRC Cells to FOLFIRI Independent of EREG Treatment

To elucidate a potential sensitization to FOLFIRI by p130Cas inhibition and the effects of parallel EREG treatment, Caco2, Lovo, and MCF7 cells were transfected with p130Cas or control siRNA and subsequently treated with recombinant EREG (Figure 4E). A significantly reduced FOLFIRI IC_50_ was measured after p130Cas knockdown in Caco2 (p130Cas siRNA IC_50_ 205 nM vs. control siRNA 460 nM; *p* < 0.05) and Lovo (1.53 µM vs. 2.26 µM; *p* < 0.001) cells, which was not observed in MCF7 cells. EREG treatment did not influence the response to FOLFIRI, neither in control nor in p130Cas siRNA transfected cells. These data suggest that p130Cas reduces FOLFIRI efficacy and thus may represent a therapeutic target in CRC.

### 2.7. p130Cas Is Involved in Controlling Cytochrome P450 Drug Metabolism and Epithelial-Mesenchymal Transition (EMT) and MTORC Signaling Pathways

**Figure 5:**
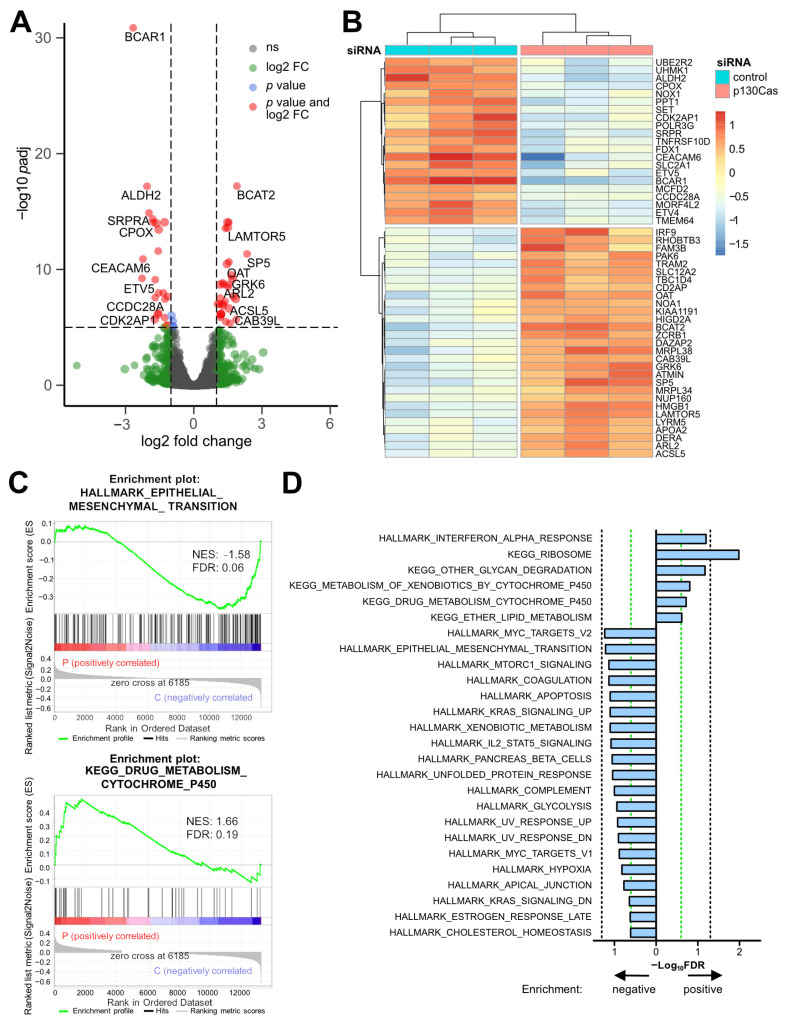
p130Cas is involved pathways controlling epithelial-mesenchymal transition (EMT), MTORC signaling, and cell/drug metabolism. (**A**–**D**). Caco2 cells were transfected with either p130Cas or control siRNA. After 48 h, RNA was isolated, and subsequently, the transcriptome was analyzed by poly(A) RNA sequencing. (**A**) Volcano plot displaying differentially expressed genes (DEGs) in p130Cas knockdown vs. control cells. (**B**) Unsupervised hierarchical clustering showing the expression changes of the top 50 DEGs in three independent transfection experiments. (**C**,**D**) GSEA of p130Cas knockdown (P) vs. control (**C**) cells. FDR, false discovery rate. NES, normalized enrichment score. (**D**) Displayed are −log_10_ FDR of the indicated genesets. Bars to the right indicate positive, bars to the left negative enrichment. Black dashed lines, FDR = 0.05. Green dashed lines, FDR = 0.25.

To start understanding how p130Cas modulates genes and cellular programs contributing to cancer progression as well as the observed induced FOLFIRI resistance in CRC, a transcriptome analysis was performed. Caco2 cells were transfected with p130Cas or control siRNA, and the isolated mRNA was subsequently subjected to poly(A) RNA sequencing. Knockdown of *BCAR1* and the previously found *EREG* upregulation in Caco2 cells were confirmed (Figure S4B). A total of 194 differentially expressed genes (DEGs; log_2_ fold change ≥ 1; *p* adjusted (*p_adj_*) ≤ 0.05) were identified in p130Cas knockdown cells (Figure 5A, Table S5). Within the top 50 DEGs, various genes previously connected with drug metabolism/resistance (e.g., *ALDH2*), cell cycle (e.g., *CDK2AP1*), MTORC signaling (e.g., *LAMTOR5*), and Wnt/β-catenin signaling (e.g., *SP5*), as well as the CRC biomarker CEACAM6, were influenced by reduced p130Cas levels (Figure 5B). Correspondingly, gene ontology analysis revealed that the p130Cas expression signature is associated with gene set enrichment analysis (GSEA) data sets such as HALLMARK_EPITHELIAL_MESENCHYMAL_TRANSITION (normalized enrichment score (NES): −1.58; *p_adj_* 0.06) and KEGG_DRUG_METABOLISM_CYTOCHROME_P450 (NES: 1.66; *p_adj_* 0.19) (Figure 5C). Moreover, associations with MTORC1-, KRAS-, and IL2/STAT5-signaling, as well as multiple genesets correlated with cell and drug metabolism, were found (Figure 5D). These results suggest that p130Cas drives CRC progression and FOLFIRI resistance by activating EMT and MTORC signaling pathways and controlling the cytochrome P450 drug metabolism.

### 2.8. BCAR1 Expression Is Associated with Poor Outcome in Right-Sided, Stage I/II, MSS or BRAF-Mutated CRC

**Figure 6:**
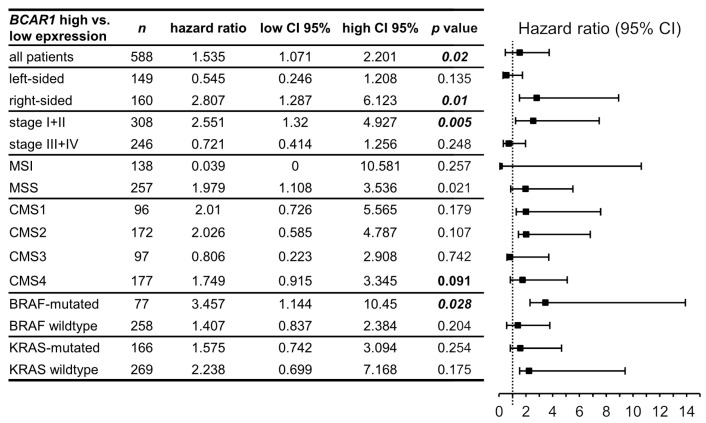
*BCAR1* expression is associated with poor outcomes in right-sided, stage I and II, MSS, or *BRAF*-mutated CRCs. Overall survival was analyzed in the indicated subgroups (TCGA CoAd data set) as described in Materials and Methods. CI, confidence interval.

To identify subtypes of CRC that might benefit from targeting p130Cas survival, analyses were extended to different subgroups in the TCGA CoAd data set based on localization, stage, consensus molecular subtype (CMS), as well as MSI and *BRAF*/*KRAS* status (Figure 6). High *BCAR1* expression was significantly associated with worse outcome in right-sided (*p* = 0.01, HR = 2.807), stage I/II (*p* = 0.005, HR = 2.55), MSS (*p* = 0.021, HR = 1.979) or *BRAF*-mutated (*p* = 0.028, HR = 3.457) tumors. No association was found regarding *KRAS* status. A trend was observed for CMS4 (*p* = 0.091, HR = 1.749). These data show that *BCAR1* may represent a prognostic marker in certain subgroups of CRC.

## 3. Discussion

Only a few studies addressed the role of p130Cas/*BCAR1* and especially its clinical implications in CRC. Van Slambrouck et al. showed that the FAK-Src-p130Cas-JNK pathway was activated and required for α1-integrin-mediated invasion of a CRC cell line [24]. Moreover, phosphorylation of p130Cas tyrosine residue Y128 was regulated by protein tyrosine phosphatase non-receptor 14 (PTPN14) [25]. p130Cas Y128 phosphorylation was important for in vitro migration, colony formation, AKT activity, and in vivo tumor growth of CRC cell lines. In addition, CRC cell lines with high levels of p130CAS Y128 phosphorylation were more sensitive to the Src family inhibitor Dasatinib [25]. The same research group could show in a study focusing on paxillin and protein tyrosine phosphatase receptor T (PTPRT) in CRC that phosphorylated Y88 in paxillin impacts the AKT pathway by controlling the interaction of the p85 regulatory PI3K subunit and p130Cas [26]. In a recent study, cysteine-rich protein 2 (CSRP2) was shown to inhibit p130Cas phosphorylation and subsequent Rac family small GTPase 1 (Rac 1) activation, thereby increasing the activity of the Hippo pathway and decreasing the p21-activated kinase (PAK)-cortactin and MAPK pathways [27]. This resulted in suppressed EMT in vitro and reduced tumorigenicity and metastasis of CRC in vivo. These studies clearly indicated the importance of p130Cas as an integral part of multiple signaling pathways involved in CRC aggressiveness. Due to the addiction of CRC on both KRAS/MAPK/ERK- and PIK3CA/AKT-signaling pathways and the important role of p130CAS as an integrator of signaling pathway activities, we were interested in a possible role of p130Cas as a biomarker as wells as a target for CRC-specific chemotherapy in CRC. Therefore, the aim of this study was to expand our understanding of p130Cas as a potential prognostic CRC marker, its role in therapy efficacy, and thus as a therapeutic target in CRC.

*BCAR1* expression was significantly higher in the CoAd TCGA data set than in the breast invasive carcinoma set. This is of special interest as *BCAR1* was initially identified as overexpressed in breast cancer, where it was associated with poor outcome and therapy resistance [14,37]. The even higher expression in CoAd suggests a similar involvement. Indeed, high *BCAR1* expression was significantly associated with reduced overall survival. Comparable p130Cas expression and phosphorylation were observed in a panel of CRC and two breast cancer cell lines, which was not correlated with MSI nor mutational status. Active (phosphorylated) p130Cas is a key driver of migration and control of proliferation in breast cancer [14]. Correspondingly, p130Cas also controlled migration in CRC cells. Unexpectedly, proliferation was stimulated in the absence or at reduced expression of p130Cas (induced by *BCAR1* knockdown). This suggests that p130Cas limits the activity or expression of, e.g., growth factors. Therefore, a TCGA database search for growth factors involved in EGFR signaling correlated with *BCAR1* expression was performed because EGFR drives proliferation and is a target for EGFR-directed antibody treatment in many carcinomas (especially CRC) [32]. This search revealed an inverse correlation of the expression of *BCAR1* and the EGF family member epiregulin (*EREG*). Accordingly, RNA interference experiments indicated that *BCAR1* and *EREG* limit each other’s expression in CRC cells. Moreover, recombinant EREG decreased p130Cas phosphorylation and *BCAR1* levels. Since p130Cas controls its own expression through a positive feedback loop [35], reduced phosphorylation may subsequently lead to lower *BCAR1*/p130Cas levels, which in turn prevents the inhibition of EREG expression by p130Cas signaling. How *BCAR1*/*EREG* levels are balanced/maintained in CRC is currently unknown. Other factors/mechanisms, such as enhanced integrin signaling [13], increasing c-Src activity in colorectal tumorigenesis [38], or alterations in the early growth response (EGR1)/NGFI-A binding protein (NAB2)/p130Cas network [23,35,39] may lead to constitutively active p130Cas limiting EREG expression. A comprehensive analysis according to stage and localization discovered that this inverse correlation was more pronounced in earlier stage and right-sided CRC. The increased right-sided negative correlation can in part be explained by the significantly lower median *EREG* levels compared with left-sided tumors, whereas *BCAR1* expression was not significantly different. Nevertheless, median expressions do not reflect the relationship of *BCAR1*/*EREG* expression in single samples as Spearman correlation coefficients do. Interestingly, a switch to a positive *BCAR1*/*EREG* correlation was observed in stage IV rectal and sigmoidal cancers. Since no global expression changes occurred from stage I–IV, the loss of the negative correlation is probably attributed to a disconnection of *BCAR1* and *EREG* expression. This disconnection is further supported by a positive correlation of both expressions in metastases (GSE121418), which coincided with the upregulation of *BCAR1* and *EREG* to equal levels. The observed disconnection of *BCAR1* and *EREG* in late-stage left-sided primary tumors and metastases points to the involvement of other factors/mechanisms influencing their expression, such as methylation changes within the *EREG* promoter [40] or altered cross-signaling through interactions with the tumor microenvironment [41]. In addition, expression levels are influenced by extracellular stimuli and even therapeutic drugs such as p130Cas by phorbol esters [35,39] or thymidylate synthase (TYMS) by 5-FU [42]. Addressing this, we could show that *BCAR1* was induced by both 5-FU and FOLFIRI in cell lines and that *EREG* levels were significantly increased in post-treatment pulmonary metastases. Moreover, standard treatment led to a greatly enhanced co-expression of *BCAR1*/*EREG* in metastases but not in primary tumors and even a switch from a negative (*r_s_* − 0.37) to a positive (*r_s_* + 0.56) correlation in pulmonary metastases.

*BCAR1*/p130Cas drives treatment resistance in breast cancer and other malignancies [13], but EREG expression is associated with suitable outcomes, especially in left-sided mCRC [33,34]. Experiments investigating the impact of *BCAR1* and EREG on chemotherapy showed that reduction of *BCAR1*/p130Cas sensitizes CRC cells to FOLFIRI, whereas EREG had no effect itself and on p130Cas-mediated effects. This suggests that p130Cas plays a functional/biological role in FOLFIRI response and may therefore be a therapeutic target in CRC patients while the impact of EREG besides as a prognostic factor remains elusive. Of note, FOLFIRI response was not altered by p130Cas knockdown in MCF7 breast cancer cells, indicating distinct mechanisms modulating p130Cas-mediated therapy response in CRC and breast cancer. This was supported by our PhosphoSitePlus analysis showing that, in part, different phosphorylation motifs are used in both cancer types.

To elucidate mechanisms by which p130Cas contributes to CRC malignancy and might limit chemotherapy efficacy, transcriptome analyses followed by gene ontology and differential gene expression analyses after p130Cas knockdown were conducted. *BCAR1* was associated with GSEA data sets and differentially expressed genes (DEGs) involved in EMT, Wnt-, and MTORC1 signaling, as well as drug metabolism. The KEGG_DRUG_METABOLISM_CYTOCHROME_P450 signature was enriched in *BCAR1* knockdown cells. Members of the cytochrome P450 (CYP) family are required for the conversion of various cancer prodrugs to their active metabolites, such as irinotecan, tegafur (5-FU prodrug), tamoxifen, and cyclophosphamide [43,44]. Among the top 50 DEGs, the cancer stem cell marker aldehyde dehydrogenase 2 (ALDH2), associated with drug efflux/resistance [45], was down-regulated after *BCAR1* knockdown. These results imply that p130Cas mediates the observed reduced FOLFIRI efficacy in part by being involved in signaling pathways controlling CYP family members and ALDH2 expression. Moreover, the HALLMARK_EPITHELIAL_MESENCHYMAL_TRANSITION signature was negatively enriched after *BCAR1* knockdown. Accordingly, p130Cas was a key driver of migration in our experiments. p130Cas was previously shown to be a key regulator of TGF-β-mediated EMT in oral squamous cell carcinoma [22] and a negative regulator of E-cadherin in breast cancer [46]. In CRC, cysteine-rich protein 2 (CSRP2) suppresses EMT via limiting p130Cas phosphorylation resulting in a switch to HIPPO signaling [27]. These studies suggest that p130Cas promotes CRC by participating in the EMT program. In addition, p130Cas signaling seems to control carcinoembryonic antigen-related cell adhesion molecule 6 (CEACAM6) expression. CEACAM6 belongs to the long-standing CEACAM CRC biomarker family, and its overexpression drives CRC invasion [47]. As observed for *BCAR1*, the highest CEACAM6 expression was found in metastases [47]. Overexpression of CEACAM6 leads to increased c-Src activity [47], which may enhance p130Cas phosphorylation and activate the p130Cas feedforward loop [35], resulting in a constitutive expression of both proteins.

The involvement of p130Cas in drug resistance/metabolism, EMT, and migration emphasizes its potential as a therapeutic target. Moreover, its distinct expression correlation with EREG at different CRC stages and origin suggests a different prognostic role in CRC subgroups/stages. Indeed, significant associations with poor outcomes were found in *BCAR1* expressing right-sided, stage I/II, MSS, or *BRAF*-mutated CRC, indicating that these subgroups might benefit from p130Cas inhibition. Unfortunately, its prognostic role in metastases could not be investigated due to missing survival data and consequently low statistical power. The observed low *EREG* levels and the strong inverse *BCAR1*/*EREG* correlation in right-sided tumors might explain the worse outcome in right-sided early-stage CRC. Our results suggest that in cooperation with other currently unknown factors/mechanisms, *BCAR1*/p130Cas may prevail over EREG in right-sided tumors. This may lead to impaired EREG signaling and therefore increased p130Cas activity. On the one hand, this may result in continuously limited EREG expression and, on the other hand, in p130Cas-driven CRC progression. These findings might help to explain the previously described lower *EREG* expression and thus poorer response to standard treatments in right-sided mCRC [33,34]. Future studies have to explore the prognostic value of *BCAR1*/p130Cas expression as well as a combined *BCAR1*/p130Cas+*EREG* signature score and its potential application in routine diagnostics (preferably measured by immunohistochemistry (IHC)). The feasibility of EREG IHC scoring even by artificial intelligence-assisted IHC evaluation was recently shown [48].

In conclusion, *BCAR1*/p130Cas may represent a prognostic marker in CRC subtypes/stages and a therapeutic target. The identified *BCAR1*/*EREG* expression correlations might be useful in future clinical patient management. However, their clinical roles at different cancer stages or in tumors of distinct origin need to be confirmed in larger prospective clinical trials.

## 4. Materials and Methods

### 4.1. Generation of Expression Constructs

Generation of the pCXbsr expression construct for human full-length *BCAR1*/p130Cas was described previously [23]. To establish a transfection control expression plasmid, the chloramphenicol acetyl transferase (*CAT*) gene was subcloned into the pCXbsr vector. Therefore, the *CAT* gene was amplified by polymerase chain reaction (PCR) followed by use of the In-Fusion HD Cloning Kit (TaKaRa, Saint-Germain-en-Laye, France) according to the manufacturer’s instructions. Primers are displayed in Table S1. The construct was validated by sequencing.

### 4.2. Cell Lines and Transient Transfections

All cell lines were obtained through exchange or from CLS (Cell Lines Service, Eppelheim, Germany) and DSMZ (German Collection of Microorganisms and Cell Cultures, Braunschweig, Germany). Cells were cultured in DMEM supplemented with 10% fetal bovine serum (FBS), 1 mM sodium pyruvate, 2 mM L-glutamine, 100 U/mL penicillin, and 100 U/mL streptomycin and regularly tested for mycoplasma contaminations using the Mycoplasma Test Kit from Applichem (Darmstadt, Germany). The identity of cell lines was recurrently confirmed by an in-house short-tandem-repeat (STR) profile analysis. Transient plasmid transfection of cells was carried out using FuGENE 6 Transfection Reagent (Promega, Fitchburg, WI, USA) as recommended by the manufacturer. Expression reconstitution experiments were performed as follows: (1) Transfection of siRNA pools and four hours later (2) transfection of expression plasmids. At 24 h of transfection, cells were detached and used for subsequent migration assays as well as parallel expression analyses (at 48 h).

### 4.3. RNA Interference

Transfection of gene-specific short-interfering RNAs (siRNAs) and control siRNA (sc-37007, Santa Cruz Biotechnologies, Dallas, TX, USA) (each 20 pmol) was performed in 6-well plates with Lipofectamine RNAiMAX Transfection Reagent (Thermo Fisher Scientific, Waltham, MA, USA) according to the manufacturer’s recommendations. Depletion of human *BCAR1*/p130Cas was achieved by transfection of pooled siRNA (Santa Cruz Biotechnologies, Dallas, TX, USA, sc-36141). ON-TARGETplus SMART pool siRNA targeting EREG was purchased from Dharmacon (L-011268-00-0020; Lafayette, CO, USA).

### 4.4. Reverse Transcriptase (RT) qPCR Expression Analysis

RNA was extracted using the RNeasy kit for cell lines (Qiagen, Hilden, Germany). Total RNA (1 µg) was transcribed into cDNA using Random Hexamer Primer and the RevertAid^TM^ Reverse Transcriptase kit (both Thermo Fisher Scientific, Waltham, MA, USA). *BCAR1* and *EREG* expression was analyzed by qPCR using primers, and UPL (universal probe library) probes (Roche, Basel, Switzerland) displayed in Table S1 and the LightCycler^®^ 480 Probes Master mix (Roche, Basel, Switzerland). *BCAR1* primers were designed to amplify all N-terminal [23] and known splice variants. qPCR mixes were analyzed on a Bio-Rad^®^ CFX Connect^TM^ Real-Time PCR Detection System with the Bio-Rad^®^ CFX Manager^TM^ Software 3.1 (Bio-Rad Laboratories, Hercules, CA, USA). *GAPDH* was used for the normalization of genes of interest expression. Similar PCR efficiencies (>95%) were achieved for all investigated genes.

### 4.5. Immunoblot (IB) Analysis

Whole-cell extracts were prepared and analyzed by immunoblotting as described [35]. Briefly, cells were incubated in RIPA buffer (#9806S, Cell Signaling Technology, Danvers, MA, USA) supplemented with phosphatase (PhosSTOP) and protease (cOmplete^TM^ ULTRA Tablets) inhibitor cocktails (both Carl Roth GmbH, Karlsruhe, Germany) for 30 min on ice followed by centrifugation at 16,000× *g* and 4 °C for 30 min. Equal amounts of protein were subjected to SDS-PAGE. Imaging of Western blot membranes was conducted with a Li-COR Odyssey FC System followed by densitometric analysis of protein bands with Image-Studio lite 5.2 (Li-COR, Lincoln, NE, USA). Equal adjustments of contrast and brightness of scanned membrane images were applied to all parts of the image. The following antibodies were used: Sigma-Aldrich (St. Louis, MO, USA): β-Actin (#A-5316); Cell Signaling Technology (Danvers, MA, USA): phospho-ERK1/2 Thr^202^/Thr^204^ (#4370), total ERK1/2 (#4695), phosphospecific anti-p130Cas tyrosines, 165 (#4015), 249 (#4014), and 410 (#4011); BD Transduction Laboratories (Franklin Lakes, NJ, USA): total p130Cas (clone 21).

### 4.6. Migration Assay

Migration assays were conducted as described previously [23]. Briefly, transiently transfected cells (1 × 10^5^) were seeded in low FBS (0.5%) DMEM in the upper compartments of a Transwell chamber (6.5 mm diameter membranes, 8 µm pore size; Corning, Corning, NY, USA). High FBS (20%) medium was added to the lower compartment as chemoattractant. Migration of the cells to the underside of the filter was evaluated at 48–72 h (depending on the cell line) by staining with crystal violet and quantified by manual counting of the cells as well as with ImageJ (1.49 v) in three random fields per experiment. Similar results were obtained with both methods. Three independent experiments were performed each, in triplicates.

### 4.7. Proliferation and Cell Viability Assay

For proliferation and FOLFIRI (folinic acid, fluorouracil, irinotecan) sensitivity measurements, cells were transfected with siRNA targeting *BCAR1* or control siRNA as described above. After 24 h, cells were detached and seeded in quadruplicates at 1.5–3 × 10^3^ cells per well in 96-well plates for subsequent treatment and in 6-well plates for confirmation of p130Cas knockdown.

Proliferation was measured with or without the addition of recombinant EREG (12.5 ng/mL, Peprotech, Hamburg, Germany) after 24 h (day 0) for additional four days by AlamarBlue assay. Briefly, 10 µL AlamarBlue^TM^ reagent (Thermo Fisher Scientific, Waltham, MA, USA) was added to each well at indicated time points, and fluorescence was measured after 4 h using a Varioskan plate reader (Thermo Fisher Scientific, Waltham, MA, USA). Presented are fold values of day 0. Adj. *p*-values were calculated using the CGGC permutation test [31] for the test statistic mean T from experiments set up in quadruplicates.

FOLFIRI sensitivity was determined by treatment of p130Cas siRNA transfected cells with increasing FOLFIRI concentrations. FOLFIRI components were obtained from the pharmacy of the University Hospital of the LMU. FOLFIRI or vehicle control was added 24 h later to cells seeded in 96-well plates at increasing concentrations. Moreover, recombinant EREG (12.5 ng/mL) or vehicle control was applied. Cells were treated for 48 h before determining cell viability with the AlamarBlue assay. Cell viability and half-maximal inhibitory concentrations (IC50) were calculated using GraphPad Prism (v.8.2.1, GraphPad Software, Inc., San Diego, CA, USA). Statistical differences between dose-response curves were estimated by a one-way ANOVA and Bonferroni’s multiple comparison post-test approach (Graphpad Prism).

### 4.8. Data Sources and Statistical Analyses

mRNA expression data, mutational status, and related clinical information of the cancer genome atlas (TCGA) cohorts (PanCancer Atlas data sets) were obtained from the cBioPortal (www.cbioportal.org accessed on 31 March 2021). Expression statistics and expression correlation analysis (Spearman’s test) were performed with Graphpad Prism 8. The number of studies detecting phosphorylation of motifs in p130Cas in different cancer types by proteomics was retrieved from PhosphoSitePlus [30] (www.phosphosite.org accessed on 31 March 2021).

Overall survival (OS) was estimated with the Kaplan–Meier method and compared with logrank test (ggplot2 and survival R packages). The effect of molecular markers was estimated with the Cox proportional hazards model (survminer R package). To identify an optimized threshold value to discriminate high from low expression, the maximum sensitivity and specificity of logarithmic expression data were calculated using a receiver operator characteristic (ROC) model (R package ggplot2, R version 4.0.2). All *p*-values < 0.05 (two-sided) were regarded significant.

### 4.9. RNA Sequencing, Data Processing, and Analyses

Detailed information on the RNA sequencing protocol and subsequent quality controls, normalization, and expression analyses can be found in the Supplementary Materials and Methods. Briefly, poly(A) RNA sequencing was performed with libraries prepared with the NexteraXT kit on an Illumina Nextseq 500 (both Illumina, San Diego, CA, USA). Expression data were analyzed using R studio 3.5.3/4.0.2, Graphpad Prism 8 (GraphPad Software, Inc., San Diego, CA, USA), and GSEA (gene set enrichment analysis) [49] software.

## Data Availability

The RNA sequencing data presented in this study are available on request from the corresponding author.

## Supplementary Materials

The following are available online at https://www.mdpi.com/article/10.3390/ijms222212364/s1.
