# Supplementary material for: p130Cas Is Correlated with EREG Expression and a Prognostic Factor Depending on Colorectal Cancer Stage and Localization Reducing FOLFIRI Efficacy
Source: Int J Mol Sci. 2021 Nov 16;22(22):12364. doi: 10.3390/ijms222212364 (PMC8625396; doi:10.3390/ijms222212364)
Supplement: Supplementary file 1 [file ijms-22-12364-s001.zip › Supplementary Figures Methods proofs.pdf]

Supplementary Figures and Material and Methods

p130Cas Is Correlated with EREG Expression and a Prognostic Factor Depending on Colorectal Cancer Stage and Localization Reducing FOLFIRI Efficacy

Jörg Kumbrink[1,2,*], Pan Li [1], Agnes Pók-Udvari [1], Frederick Klauschen [1,2], Thomas Kirchner [1,2] and Andreas Jung [1,2]

1 Faculty of Medicine, Institute of Pathology, Ludwig-Maximilians-University Munich, 80337 Munich, Germany.
2 German Cancer Consortium (DKTK), partner site Munich, 803367 Munich, Germany.
* Correspondence: Joerg.Kumbrink@med.uni-muenchen.de

Figure S1

Figure S2

Figure S3

Figure S4

Table S1

Table S2

Table S3

Table S4

Supplementary Material and Methods

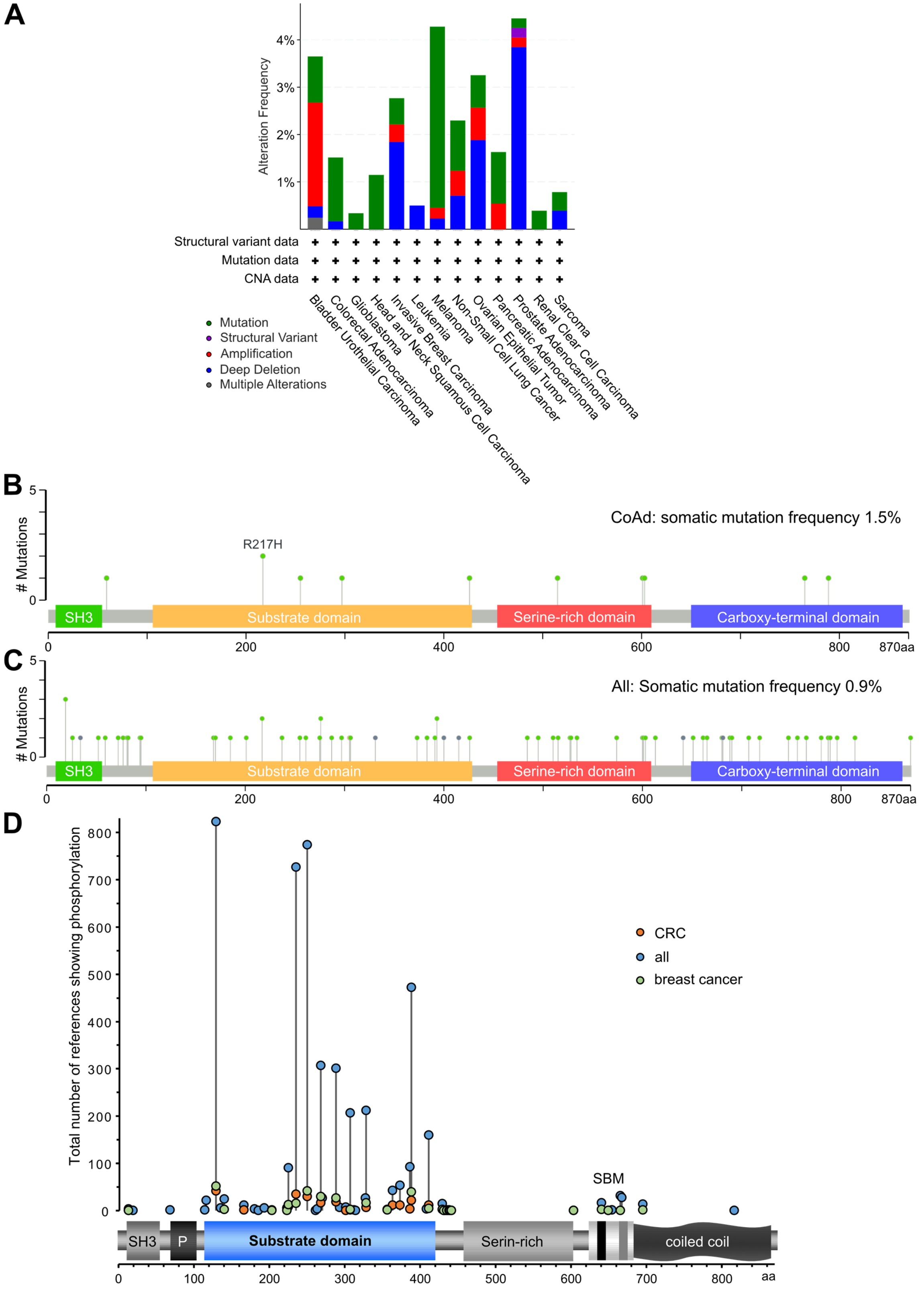

Figure S1. ***BCAR1*/p130Cas is not commonly mutated but phosphorylated in human colorectal adenocarciomas.** A. *BCAR1* alteration frequencies in the indicated TCGA PanCancer Atlas datasets. B-C. Number of reported mutations at the cBioportal in different p130Cas domains in all TCGA datasets indicated in A (B) and colorectal adenocarciomas (C). D. Number of studies showing phosphorylation of certain motifs in the different p130Cas domains as curated at PhosphositePlus. P, proline-rich domain. SBM, bipartite Src binding motif.

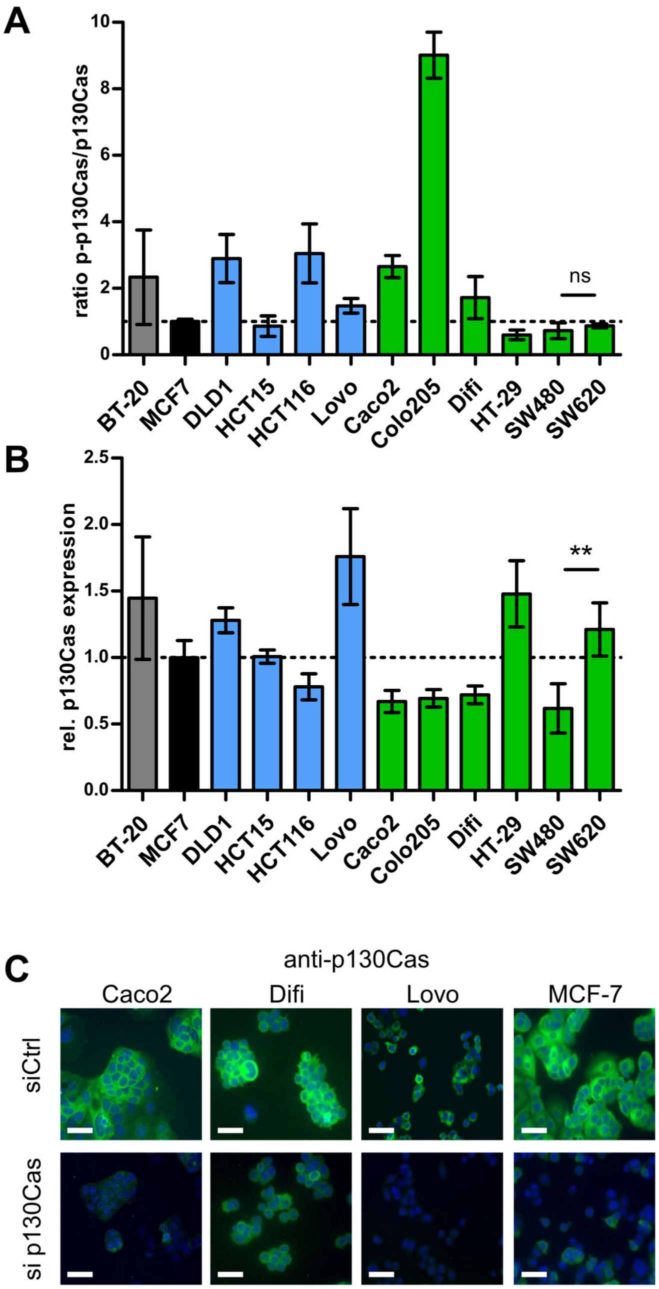

Figure S2. p130Cas expression and phosphorylation in a panel of CRC and breast cancer cell lines. A. Ratio of active p-p130Cas to p130Cas in relation to MCF7 (set to 1). **B.** Ratio of p130Cas to Actin in relation to MCF7 (set to 1). Ns, not significant. **, $p < 0.01$. **C.** Cells were transfected with siRNA targeting p130Cas or control siRNA and at 48 h p130Cas expression was analyzed by immunofluorescence staining. Scale bars, 100 μm.

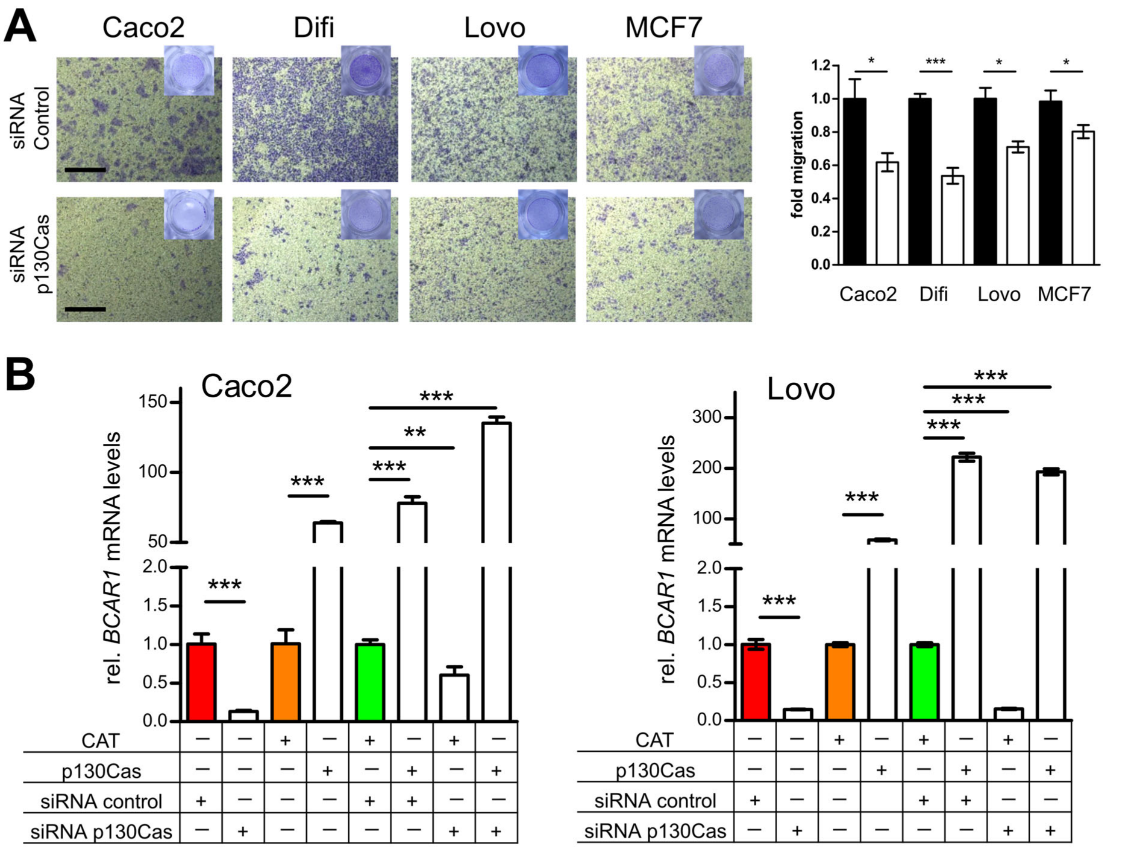

Figure S3. p130Cas drives migration in CRC cell lines. **A.** Cells transiently transfected with siRNA control or siRNA directed against p130Cas were subjected to serum-stimulated migration assay as described. Left panels, shown are representative images from at least two independent experiments. scale bars, 500 µm. Right panel, shown are fold migration (siRNA control was set to 1) and SEM of triplicates from at least two independent experiments. **B.** *BCAR1* mRNA expression levels in Caco2 and Lovo cells used for the rescue migration experiments in Figure 2E after transfection with the indicated plasmids or siRNAs. *, $p < 0.05$; **, $p < 0.01$; ***, $p < 0.001$.

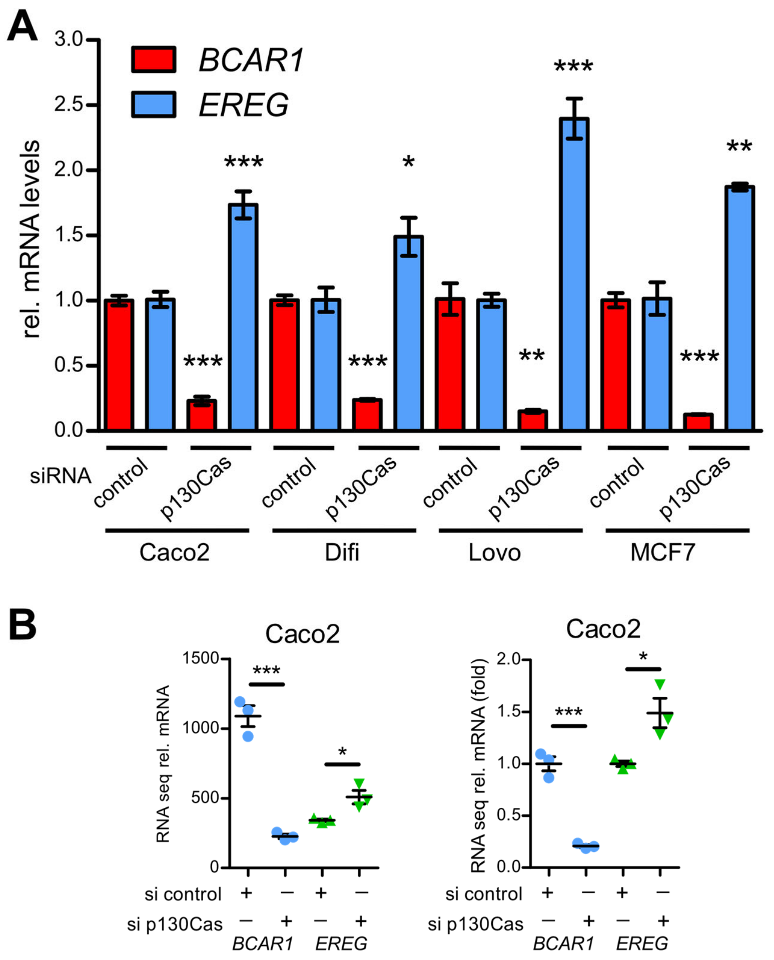

Figure S4. p130Cas/*BCAR1* knockdown induces *EREG* expression. A-B. Cells were transfected with siRNA targeting *BCAR1* or control siRNA and harvested at 48 h. *BCAR1* and *EREG* levels after p130Cas knockdown were quantified by RT qPCR (**A**) or RNA sequencing (**B**). **A.** Shown are expression levels in relation to control siRNA (set to 1) from one representative out of three performed experiments. **B.** Presented are normalized expression values (DESeq2, left panel) or expression in relation to control siRNA (set to 1, right panel) from three biological replicates. *, $p < 0.05$. **, $p < 0.01$. ***, $p < 0.001$. si, short interfering RNA.

Table S1. Primer sequences

Gene	Forward (5'−3')	Reverse (5'−3')	Purpose	UPL-#
CAT (BamHI)	CTAGACTGCCGGATCCATGGA GAAAAAAATCACTGG	GCTTAATTAAGGATCCTTACG CCCCGCCCTGCCACTCATC	Subcloning into pCXbsr	–
BCAR1	ACAGGGGAAGGAGGAGTTTG	GTGATGCTGCCCTTTTCC	mRNA expression	17
EREG	TGGTCTCTTCACTCAGGTCTCA	CGTGAGTTGGCATAGGGAAC	mRNA expression	86
GAPDH	AGCCACATCGCTCAGACAC	GCCCAATACGACCAAATCC	Expression normalization	60

Table S2. MSI and mutation status of used cell lines. In grey, cell lines were derived from the same patient.

cell line	tumor	status	RAS	BRAF	PIK3CA	CTNNB1	APC	TP53
DLD1	colon	MSI	mut	WT	mut	WT	mut	mut
HCT15	colon	MSI	mut	WT	mut	WT	mut	mut
HCT116	colon	MSI	mut	WT	mut	mut	WT	WT
Lovo	colon, distant lymph node	MSI	mut	WT	WT	WT	mut	WT
Caco2	colon	MSS	WT	WT	WT	mut	mut	mut
Colo205	caecum	MSS	WT	mut	WT	mut	mut	mut
Difi	rectum	MSS	WT	WT	WT	WT	mut	mut
HT29	colon	MSS	WT	mut	WT	WT	mut	mut
SW480	colon	MSS	mut	WT	WT	WT	mut	mut
SW620	lymph node metastasis	MSS	mut	WT	WT	WT	mut	mut
MCF7	breast	ER/PR+/MSSS	WT	WT	mut	(WT)	(WT)	WT
BT-20	breast	Triple-neg./MSS	WT	WT	mut	(WT)	(WT)	mut

Table S3. Spearman correlation of *BCAR1* and *EREG* expression in the TCGA CoAd (A) and GSE131418 (B) datasets. AJCC, American Joint Committee on Cancer.

A.

correlation *BCAR1* and *EREG*	stage (AJCC)	*n*	Spearman's Coeffcient (r_s)	*p* value
all	I	103	**−0.3355**	**0.0005**
	II	218	−0.2045	**0.0024**
	III	168	**−0.2951**	**0.0001**
	IV	85	−0.06721	0.5411
left sided (incl. Rectum)	I	47	−0.1852	0.2125
	II	87	−0.005577	0.9591
	III	91	**−0.3041**	**0.0034**
	IV	46	−0.01573	0.9174
Left-sided (-rectum)	I	32	−0.1012	0.5817
	II	57	−0.05218	0.6999
	III	70	**−0.2743**	**0.0216**
	IV	33	−0.07854	0.6639
Right-sided	I	34	**−0.5603**	**0.0006**
	II	79	**−0.3041**	**0.0064**
	III	49	−0.1383	0.3434
	IV	22	−0.2874	0.1947
C. sigmoideum	I	17	−0.1593	0.5414
	II	32	**−0.3178**	***0.0763***
	III	36	**−0.5042**	**0.0017**
	IV	24	0.09913	0.6449
rectum	I	15	−0.175	0.5327
	II	30	0.1017	0.5929
	III	21	**−0.3519**	0.1177
	IV	14	0.1473	0.6154
cecum	I	18	**−0.3519**	0.1521
	II	27	**−0.6093**	**0.0007**
	III	21	−0.213	0.3539
	IV	13	−0.05495	0.8585
C. ascendens	I	15	**−0.5893**	**0.0208**
	II	45	−0.1393	0.3616
	III	26	−0.08103	0.694
	IV	10	**−0.4545**	0.1912

p value
< 0.05

Coefficient (r_s)
< −0.25
< −0.45

Table S3

B.

**correlation *BCAR1* and *EREG***	**treatment**	***n***	**Spearman's Coeffcient (r_s)**	***p* value**
primary	pre and post	333	0.0038	0.95
	pre	235	−0.007	0.92
	post	98	0.061	0.55
metastasis	pre and post	184	0.29	<0.0001
	pre	56	0.094	0.49
	post	128	0.394	<0.0001
pulmonal metastasis	pre and post	43	0.383	0.011
	pre	12	−0.37	0.24
	post	31	0.563	0.0001
hepatic metastasis	pre and post	141	0.28	0.0008
	pre	44	0.241	0.1144
	post	97	0.35	0.0005

p value
< 0.05

Coefficient (r_s)
< −0.25
> 0.25
≥ 0.35
> 0.5

Table S4. Statistical comparison of the growth curves after the indicated treatment.

**Caco2**	control siRNA	control siRNA +EREG	p130Cas siRNA	p130Cas siRNA +EREG
control siRNA	x	0.5632	0.0012	<0.00001
control siRNA +EREG	0.5632	x	0.0028	<0.00001
p130Cas siRNA	0.0012	0.0028	x	0.00080
**Difi**	control siRNA	control siRNA +EREG	p130Cas siRNA	p130Cas siRNA +EREG
control siRNA	x	0.605	0.0006	0.001
control siRNA +EREG	0.605	x	0.001	0.0015
p130Cas siRNA	0.0006	0.001	x	0.7905
**Lovo**	control siRNA	control siRNA +EREG	p130Cas siRNA	p130Cas siRNA +EREG
control siRNA	x	0.269	<0.0001	0.0005
control siRNA +EREG	0.269	x	0.0005	0.0105
p130Cas siRNA	<0.0001	0.0005	x	0.05500
**MCF7**	control siRNA	control siRNA +EREG	p130Cas siRNA	p130Cas siRNA +EREG
control siRNA	x	0.0214	<0.00001	<0.00001
control siRNA +EREG	0.0214	x	0.1262	<0.00001
p130Cas siRNA	<0.00001	0.1262	x	0.00270

Adjusted p values were calculated using the CGGC permutation test for the test statistic mean T from three (Caco2, Difi, MCF7) and two (Lovo) independent experiments set up in quadruplicates. Red, not significant. Yellow, $p < 0.1$. Green, $p < 0.02$.

Supplementary Material and Methods

Immunofluorescence analysis

Transiently siRNA transfected cells were cultured in 8-well chamber slides. After fixation with 3% paraformaldehyde for 10 min, cells were immunostained with p130Cas antibody (clone 21, BD Transduction Laboratories, Franklin Lakes, NJ, USA) or IgG isotype control (Thermofisher, Waltham, MA, USA, #31903) followed by Alexa Fluor 488 Goat Anti-Mouse IgG (Molecular Probes, Eugene, OR, USA). Nuclei were labeled with 4′6-diamidino-2-phenylindole (DAPI, Thermofisher, Waltham, MA, USA). Imaging of stained chamber slides was performed with an Olympus BX53 fluorescence microscope (Olympus, Tokio, Japan) equipped with a CCD camera.

RNA sequencing, data processing and analyses

Cells were transfected with siRNAs targeting *BCAR1*/p130Cas or with control siRNAs as described in the main manuscript for 48 h. Three independent experiments were conducted. RNA was extracted using the RNeasy kit (Quiagen, Hilden, Germany). RNA integrity and quality was validated on an Agilent 2100 Bioanalyzer Instrument (Agilent, Santa Clara, CA, USA).

Poly(A) RNA sequencing was performed using an adapted version of the previously described protocol, which also contains the sequences of the used primers [1]. Briefly, 25 ng of fragmented total RNA was reverse transcribed with Maxima H Minus reverse transcriptase (Thermofisher, Waltham, MA, USA) using oligo(dT) primers containing barcode and unique molecular identifier (UMI) sequences (E3V6NEXT primers) as well as the universal adapter primer (E5V6NEXT). cDNA was purified with DNA Clean & Concentrator-5 columns (Zymo Research, Irvine, CA, USA), treated with Exonuclease I (New England Biolabs, Ipswich, MA, USA) and subsequently amplified (12 PCR cycles) using KAPA Hifi Hot Start polymerase (Roche, Basel, Switzerland) and SINGV6 primer. The PCR products were purified using AMPure beads (Beckman Coulter, Brea, CA, USA) and the purified DNA was quantified utilizing the Qubit dsDNA HS Assay Kit (Thermofisher, Waltham, MA, USA). Subsequently, the Nextera XT DNA library preparation kit (Illumina, San Diego, CA, USA) was used for rapid library preparation according to the manufacturer’s instructions, with the exception that the i5 primer was replaced by the P5NEXTPT5 primer. The quality of the library was checked by Qubit quantification and with an Aligent 2100 bioanalyzer using Agilent High sensitivity DNA chips (Agilent, Santa Clara, CA, USA). The library was denatured and diluted to 20 pM. Sequencing was performed on an Illumina NextSeq 500 using the NextSeq 500/550 high output v2 kit (Illumina, San Diego, CA, USA) for dual-indexed sequencing according to the manufacturer's instructions.

Sequencing raw data were processed as follows. Demultiplexing and conversion: bcl2fastq2. Quality control: FastQC/MultiQC (Galaxy module). FastQ to BAM (Alignment): RNAStar. Counting/Annotation: HTSeq-count/hg19 GTF/GFF File. Normalization of expression data and subsequent identification of expression changes and differentially expressed genes (DEGs) were performed with the R package DESeq2 (1.26) [2]. Adjusted p values/false discovery rates (FDR) were calculated using Benjamini and Hochberg correction [3]. Heat maps with hierarchical clustering of the top 50 significantly differentially expressed genes with a $\log_2$fold change ≥1/−1 were generated involving Pearson’s correlation, applying the R package pheatmap (1.0.12). Volcano plots were drawn with the EnhancedVolcano package (1.4.0) in R. Gene Set Enrichment Analysis (GSEA) were performed using the Hallmark and C2 KEGG data sets (Broad Institute, Cambridge, MA, USA) [4].

References

1. Magali Soumillon, D.C., Stefan Semrau, Alexander van Oudenaarden, Tarjei S. Mikkelsen. Characterization of directed differentiation by high-throughput single-cell rna-seq. *bioRxiv* **2014**.
2. Love, M.I.; Huber, W.; Anders, S. Moderated estimation of fold change and dispersion for rna-seq data with deseq2. *Genome Biol* **2014**, *15*, 550.
3. Benjamini, Y.; Hochberg, Y. Controlling the false discovery rate: A practical and powerful approach to multiple testing. *J. R. Statist. Soc.* **1995**, *57*, 289-300.
4. Subramanian, A.; Tamayo, P.; Mootha, V.K.; Mukherjee, S.; Ebert, B.L.; Gillette, M.A.; Paulovich, A.; Pomeroy, S.L.; Golub, T.R.; Lander, E.S., *et al.* Gene set enrichment analysis: A knowledge-based approach for interpreting genome-wide expression profiles. *Proc Natl Acad Sci U S A* **2005**, *102*, 15545-15550.
